# Enterobacterial common antigen repeat-unit flippase WzxE is required for *Escherichia coli* growth under acidic conditions, low temperature, and high osmotic stress conditions

**DOI:** 10.1128/aem.02595-24

**Published:** 2025-04-10

**Authors:** Saki Yamaguchi, Kazuya Ishikawa, Kazuyuki Furuta, Chikara Kaito

**Affiliations:** 1 Graduate School of Medicine, Dentistry and Pharmaceutical Sciences, Okayama University 199491 , Okayama, Japan; INRS Armand-Frappier Sante Biotechnologie Research Centre, Laval, Quebec, Canada

**Keywords:** wzxE flippase, enterobacterial common antigen, low pH, low temperature, hyperosmotic stress

## Abstract

Colanic acid and enterobacterial common antigen (ECA) are cell-surface polysaccharides that are produced by many *Escherichia coli* isolates. Colanic acid is induced under acidic, low temperature, and high-salt conditions and is important for *E. coli* resistance to these stresses; however, the role of ECA in these stresses is less clear. Here, we observed that knockout of flippase *wzxE*, which translocates lipid-linked ECA repeat units from the cytoplasmic side of the inner membrane to the periplasmic side, resulted in the sensitivity of *E. coli* BW25113 to acidic conditions. The *wzxE*-knockout mutant showed reduced growth potential and viable counts in vegetable extracts with acidic environments, including cherry tomatoes, carrots, celery, lettuce, and spinach. A double-knockout strain of *wzxE* and *wecF* (glycosyltransferase that adds the third-and-final sugar of the lipid-linked ECA repeat unit) was not sensitive to acidic conditions, with similar results obtained for a double-knockout strain of *wzxE* and *wcaJ* (glycosyltransferase that initiates colanic acid lipid-linked repeat-unit biosynthesis). The *wzxE*-knockout mutant was sensitive to low temperatures or high-salt conditions, which induced colanic acid synthesis, and these sensitivities were abolished by the additional knockout of *wcaJ*. These results suggest that lipid-linked ECA repeat units confer *E. coli* susceptibility to acidic, low temperatures, and high-salt conditions in a colanic acid-dependent manner and that *wzxE* suppresses this negative effect.

**IMPORTANCE:**

The role of the common enterobacterial antigen, a polysaccharide that is conserved throughout enterobacteria, in stress resistance is unclear. Our results suggest that lipid-linked enterobacterial common antigen repeat units (which are typically translocated across the inner membrane by the flippase WzxE) cause sensitivity of *Escherichia coli* to acidic, low-temperature, and high-salt conditions in a manner dependent on colanic acid. The *wzxE*-knockout mutant was sensitive to crude vegetable extracts, suggesting that the development of WzxE inhibitors could lead to novel food poisoning prevention agents. Considering previous findings that lipid-linked ECA repeat units are flipped by both WzxE and the flippase for colanic acid lipid-linked repeat-unit, the colanic acid-dependence of the *wzxE*-knockout phenotype proposes a model in which a large amount of colanic acid under stress conditions occupies the flippase for colanic acid lipid-linked repeat unit, leading to accumulation of lipid-linked ECA repeat units on the inner membrane.

## INTRODUCTION

According to the WHO Foodborne Disease Burden Epidemiology Reference Group (2015), 35,000 people died from enteric diseases caused by enteropathogenic *Escherichia coli* in 2010, and *E. coli*-mediated foodborne diseases remain a problem. Because *E. coli* is an enteric bacterium found in animal feces and urine, one of the most common causes of food poisoning is the contamination of crops by animal feces and manure in nature during food growth and processing stages. As an example, food poisoning caused by pathogenic *E. coli* in cut vegetables and salads has become a problem. *E. coli* is often exposed to acidic and other stresses, such as low temperatures and desiccation. Therefore, it is important to understand the mechanisms underlying the resistance of *E. coli* to these stressors.

*Escherichia coli* resists stresses such as acidic stress by covering its cell surface with polysaccharides. Polysaccharides present on the surface of *E. coli* include O and K antigens, colanic acid, and enterobacterial common antigens (ECA). Because many strains commonly produce colanic acid and ECA, it is important to investigate their roles under stress. Colanic acid is composed of a repeating trisaccharide (→3]-β-Fuc-[1→4]-α-FucOAc-[1→3]-β-Glu-[1→) with a trisaccharide side branch (β-Gal3,4Pyr-[1→4]-β-GlcA-[1→3]-β-Gal-[1→4) linked to the terminal Fuc residue (1, 2). It is secreted into the extracellular milieu as a high-molecular weight or bound to lipid A-core oligosaccharide of LPS (3). Colanic acid synthesis is enhanced by acidic conditions (4–6), low temperature (7), high-salt stress (8), and desiccation (9), suggesting that colanic acid protects *E. coli* cells under various stressful conditions. In the EHEC strain O157:H7, mutations in the *wca* operon required for colanic acid biosynthesis resulted in sensitivity to acidic conditions (4).

ECA is built with a repeating trisaccharide (→3]-α-Fuc4NAc-[1→4]-β-ManNAcA-[1→4]-α-GlcNAc-[1→) unit and has been found on the cell surface in two distinct forms: ECA_LPS_ (bound to lipid A-core oligosaccharide of LPS) and ECA_PG_ (bound to phosphoglycerol). A cyclic form of ECA (ECA_CYC_) has also been identified in the periplasm. Although ECA_CYC_ is involved in the outer membrane permeability barrier (10), other physiological roles of ECAs are poorly understood.

Both ECA and colanic acid are synthesized and secreted via distinct Wzx/Wzy-dependent pathways. In these schemes, nucleotide sugar phosphates are bound to the undecaprenyl (Und) phosphate at the cytoplasmic face of the inner membrane (IM) to form pyrophosphate (PP) linkages. Various glycosyltransferases add additional sugars to the initial monosaccharide to generate Und-PP-linked sugar-repeat units. These lipid-linked repeat units are then preferentially translocated by pathway-specific Wzx flippases from the cytoplasmic to the periplasmic face of the IM. There, they are then joined together by pathway-selective Wzy polymerases influenced by polysaccharide-*co*-polymerase proteins that contribute to polymer modal-length determination. Following this processing stage, and depending on the nature of the specific polysaccharide, sugar polymers can become (i) ligated to lipid A-core oligosaccharide to form a mature LPS molecule that is then translocated to the cell surface, (ii) secreted outside of the cell to become loosely associated with the cell surface, (iii) modified with a phosphatidyl glycerol moiety and become surface-integrated, or (iv) cyclized and freely released into the periplasmic space.

**Fig 1:**
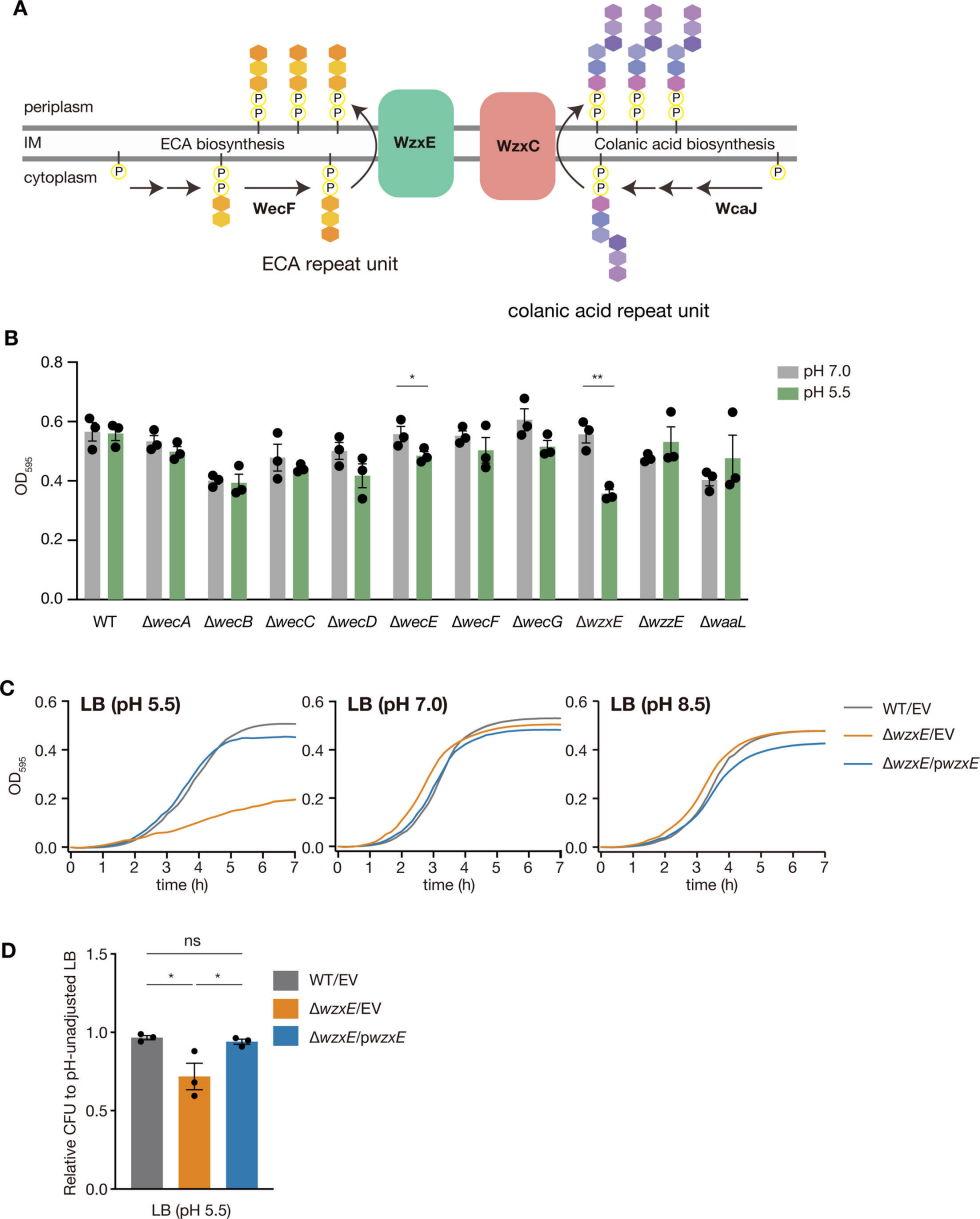
*wzxE*-knockout mutant is sensitive to acidic conditions. (**A)** Model diagram of the enterobacterial common antigen synthesis pathway and the colanic acid synthesis pathway. (**B)** *Escherichia coli* wild-type strain and the gene-knockout mutants of the ECA synthesis pathway were cultured in LB 50 mM MES (pH 5.5) and LB 40 mM MOPS (pH 7.0) at 37°C. The OD_595_ was measured at 5 h. Data represent the mean ± SE of three biological repeats. Unpaired t-test was performed (*P < 0.05, **P < 0.01). (**C)** The *E. coli* wild-type strain transformed with pMW118 (WT/EV), *wzxE*-knockout mutant transformed with pMW118 (Δ*wzxE*/EV), and *wzxE*-knockout mutant transformed with pwzxE (Δ*wzxE*/pwzxE) were cultured in LB 50 mM MES (pH 5.5), LB 40 mM MOPS (pH 7.0), or LB 50 mM Tricine (pH 8.5), and the OD_595_ was measured for 7 h at 37°C. Data represent the mean ± SE of three biological repeats. (**D)** Overnight bacterial cultures in LB medium were diluted and plated on LB agar medium or LB 50 mM MES (pH 5.5) agar medium, and the number of colonies formed was counted. The vertical axis represents the relative CFUs on LB 50 mM MES (pH 5.5) agar against those on the LB agar plate as a normal scale. Data represent the mean ± SE of three biological repeats. One-way ANOVA with Tukey’s post hoc test *P*-values is provided (*P < 0.05).

In this study, we examined the acid sensitivity of strains deficient in genes involved in ECA synthesis to understand the physiological role of ECA. Our results revealed that the *wzxE* knockout increased acid sensitivity. WzxE is a flippase that translocates Und-PP-linked ECA trisaccharide repeat units across the IM (Fig. 1A). Accumulation of these lipid-linked repeat units is lethal; however, in *E. coli wzxE*-knockout strains, lethal accumulation of Und-PP-linked ECA repeat units is prevented by inefficient flipping by the flippases WzxC (Fig. 1A) and WzxB for Und-PP-linked repeat units of colanic acid and O antigen, respectively (11–13). Although the *wzxE*-knockout strain is susceptible to nalidixic acid and amikacin (14), its phenotype in acidic environments has not been investigated. In this study, we aimed to elucidate the mechanism underlying the acid sensitivity of the *wzxE*-knockout strain.

## RESULTS

### *wzxE-*knockout strain is sensitive to acidic growth medium

We first examined whether strains deficient in genes involved in ECA synthesis were sensitive to acidic conditions and observed that the *wzxE*-knockout mutant showed reduced proliferative ability compared to the wild-type strain in LB medium adjusted to pH 5.5 (Fig. 1B and C). In contrast, the *wzxE*-knockout mutant showed no significant difference in growth in LB medium adjusted to pH 7.0 or pH 8.5 compared to the wild-type (WT) strain (Fig. 1C). Additionally, the *wzxE*-knockout mutant had fewer colony-forming units (CFUs) than the WT strain when an overnight culture grown in pH-unadjusted LB liquid medium (pH 6.7) was spread onto LB agar medium adjusted to pH 5.5 (Fig. 1D). The introduction of a plasmid carrying the *wzxE* gene into the *wzxE*-knockout mutant restored WT-like proliferative ability or CFUs at pH 5.5 (Fig. 1C and D). These results suggest that *wzxE* is required for growth under acidic conditions.

### *wzxE*-knockout strain is sensitive to acidic commercial vegetable juice

**Fig 2:**
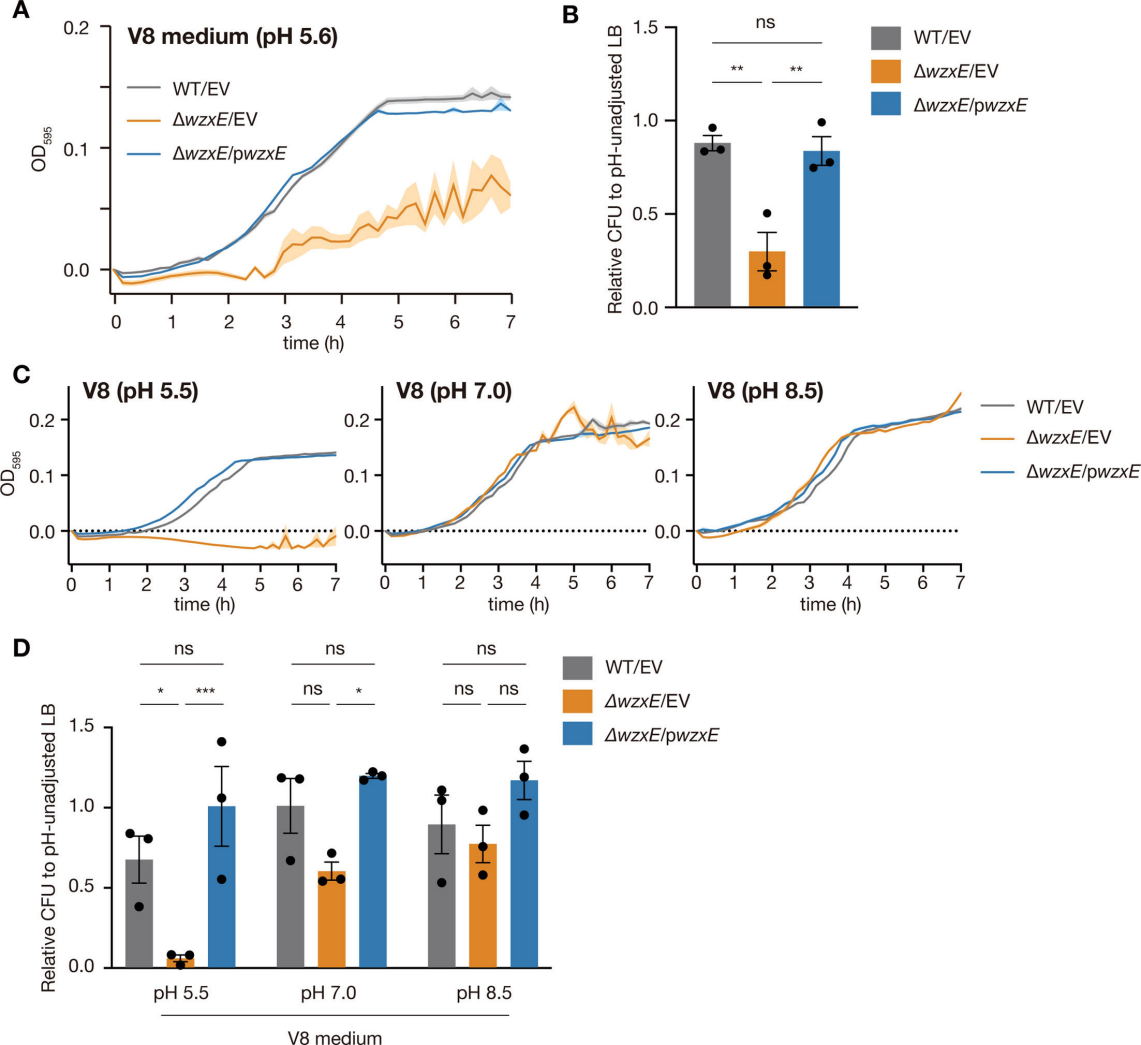
*wzxE*-knockout mutant is V8-sensitive. (**A)** *Escherichia coli* wild-type strain transformed with pMW118 (WT/EV), *wzxE*-knockout mutant transformed with pMW118 (Δ*wzxE*/EV), and *wzxE*-knockout mutant transformed with pwzxE (Δ*wzxE*/pwzxE) were grown in V8 medium, and the OD_595_ was measured for 7 h at 37°C. Data represent the mean ± SE of three biological repeats. (**B)** Overnight bacterial cultures in LB medium were diluted and plated on an LB agar plate or V8 agar plate, and the number of colonies formed was counted. The relative CFUs on the V8 agar plate against those on the LB agar plate are shown. Data represent the mean ± SE of three biological repeats. One-way ANOVA with Tukey’s post hoc test *P*-values is provided (**P < 0.01). (**C)** *E. coli* wild-type strain transformed with pMW118 (WT/EV), *wzxE*-knockout mutant transformed with pMW118 (Δ*wzxE*/EV), and *wzxE*-knockout mutant transformed with pwzxE (Δ*wzxE*/pwzxE) were cultured in V8 50 mM MES (pH 5.5), V8 40 mM MOPS (pH 7.0), or V8 50 mM Tricine (pH 8.5), and the OD_595_ was measured for 7 h at 37°C. Data represent the mean ± SE of three biological repeats. (**D)** Overnight bacterial cultures in LB medium were diluted and plated on LB agar medium, V8 50 mM MES (pH 5.5) agar medium, V8 40 mM MOPS (pH 7.0) agar medium, or V8 50 mM Tricine (pH 8.5) agar medium, and the number of colonies formed were counted. The relative CFUs against those on the LB agar plate are shown. Data represent the mean ± SE of three biological repeats. One-way ANOVA with Tukey’s post hoc test *P*-values is provided (*P < 0.05, ***P < 0.001).

In nature, plant settings are considered acidic (15). We next investigated whether the *wzxE*-knockout mutant showed reduced proliferation in V8 medium prepared from V8 vegetable juice, which is acidic at pH 5.6. V8 vegetable juice is made from tomato, carrot, celery, beets, parsley, lettuce, watercress, spinach, and other ingredients (salt, citric acid, natural flavors, vitamin C, beta carotene). In V8 medium, the *wzxE*-knockout mutant showed reduced proliferative ability compared to the WT strain (Fig. 2A). Additionally, the CFUs of the *wzxE*-knockout mutant on the V8 agar medium were significantly reduced compared to that of the wild-type strain (Fig. 2B). The introduction of a plasmid carrying the *wzxE* gene into the *wzxE*-knockout mutant restored the growth and CFUs of this strain in V8-liquid and V8-agar media (Fig. 2A and B). These results suggest that the *wzxE*-knockout mutant is sensitive to the V8 conditions.

Next, we examined whether the V8 sensitivity of the *wzxE*-knockout mutant was pH-dependent. Increasing the pH of the V8 medium to 7.0 resulted in the *wzxE*-knockout strain no longer exhibiting compromised growth or CFUs (Fig. 2C and D). These results suggest that the V8 sensitivity of the *wzxE*-knockout mutant is pH-dependent.

### *wzxE* knockout strain is sensitive to crude vegetable extracts

**Fig 3:**
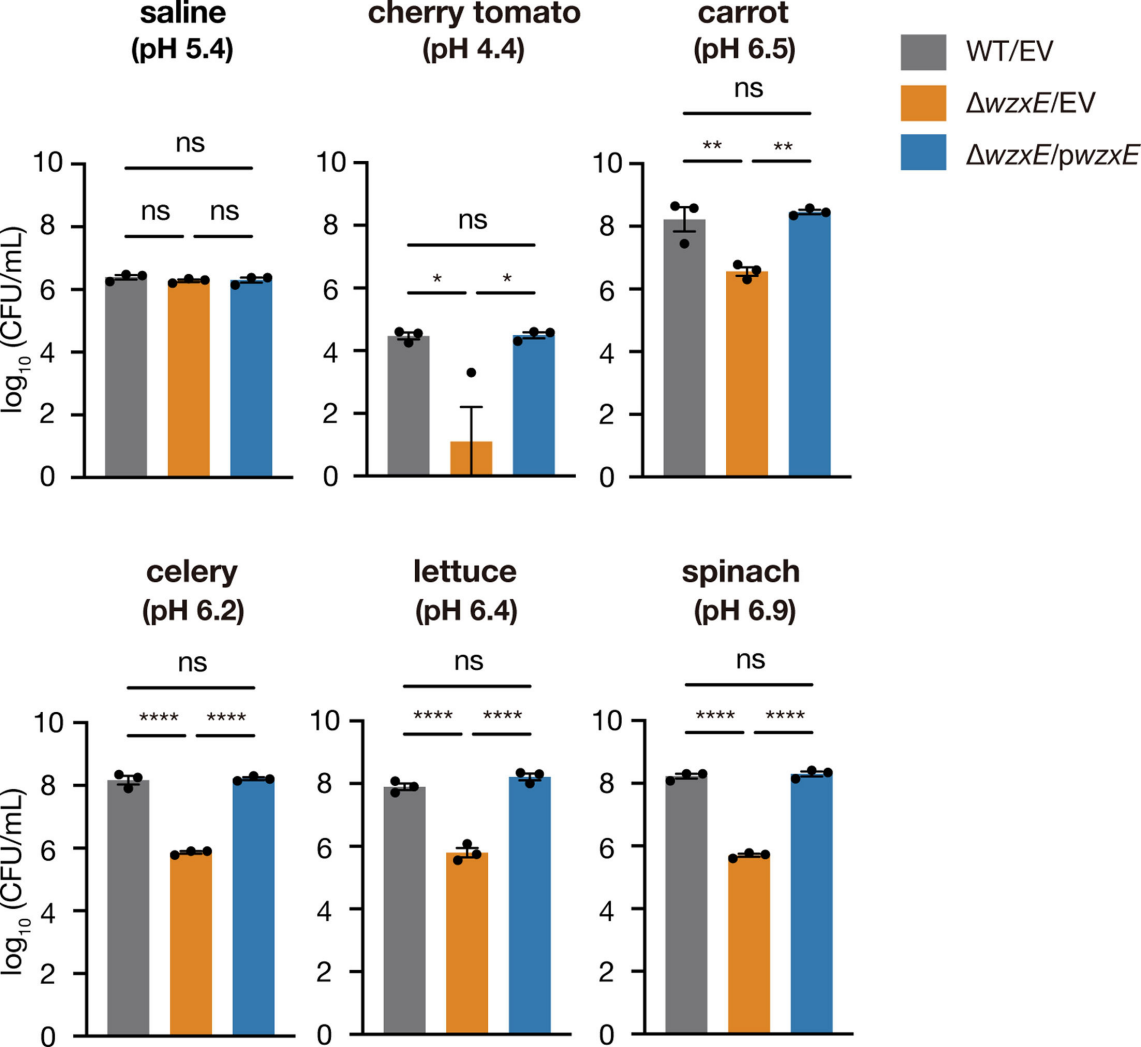
*wzxE*-knockout mutant is sensitive to vegetable crude extracts. Overnight bacterial cultures of the *Escherichia coli* wild-type strain transformed with pMW118 (WT/EV), *wzxE*-knockout mutant transformed with pMW118 (Δ*wzxE*/EV), and *wzxE*-knockout mutant transformed with pwzxE (Δ*wzxE*/pwzxE) were inoculated into saline or crude extracts of cherry tomato, carrot, celery, lettuce, or spinach, and the viable counts were determined after 24 h of incubation at 25°C. The number of viable cells is shown on the vertical axis. Data represent the mean ± SE of three biological repeats. One-way ANOVA with Tukey’s post-hoc test *P*-values is provided (*P < 0.05, **P < 0.01, and ****P < 0.0001).

Given that the *wzxE*-knockout mutant was susceptible to medium supplemented with commercially processed vegetable juice, we next determined whether the *wzxE*-knockout mutant was susceptible to raw vegetable extracts. To this end, we measured the number of viable bacteria in the crude extracts of cherry tomatoes, carrots, celery, lettuce, and spinach. The results illustrated that the *wzxE*-knockout mutant had significantly lower viable counts than the WT strain in treatments with all vegetable extracts tested (Fig. 3). In contrast, the *wzxE*-depleted mutant showed no significant difference in viable counts in saline compared to the WT strain (Fig. 3). The introduction of a plasmid carrying the *wzxE* gene into the *wzxE*-knockout mutant restored viable counts in vegetable extracts (Fig. 3). The pH values of the vegetable crude extracts used in this study were 4.4 for cherry tomatoes, 6.5 for carrots, 6.2 for celery, 6.4 for lettuce, and 6.9 for spinach. A significant reduction in viable bacterial counts was observed in cherry tomatoes, which had a particularly low pH. These results suggest that the *wzxE*-knockout mutant is susceptible to crude vegetable extracts.

### Und-PP-linked ECA repeats affect *wzxE*-knockout strain sensitivity to acidic conditions

**Fig 4:**
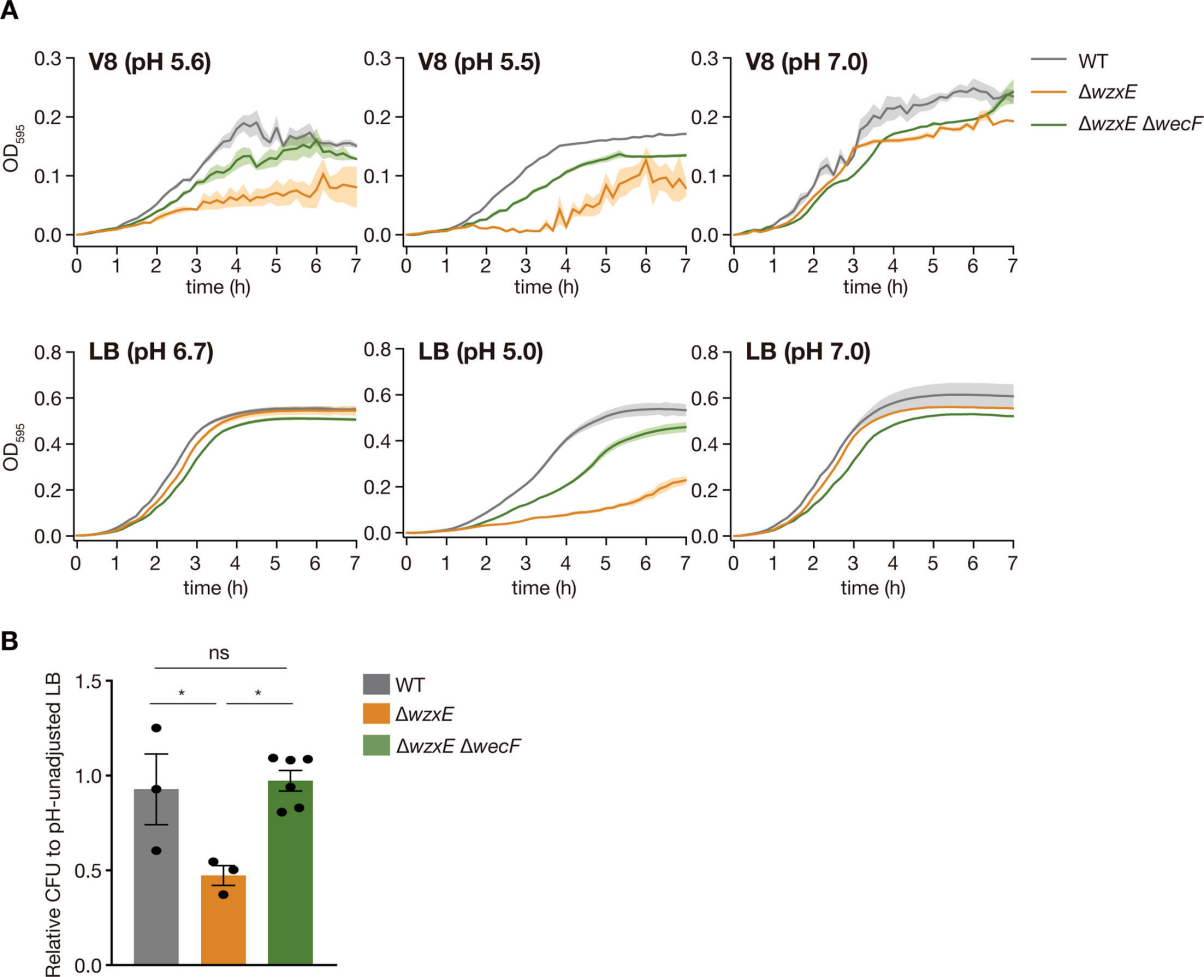
*wzxE*/*wecF* double-knockout mutant is not sensitive to acidic conditions. (**A)** *Escherichia coli* wild-type strain (WT), *wzxE*-knockout mutant (Δ*wzxE*), and *wzxE*/*wecF* double-knockout mutant (Δ*wzxE* Δ*wecF*) were cultured in V8, V8 50 mM MES (pH 5.5), V8 40 mM MOPS (pH 7.0), LB, LB 50 mM MES (pH 5.0), or LB 40 mM MOPS (pH 7.0) , and the OD_595_ was measured for 7 h at 37°C. Data represent the mean ± SE of three biological repeats. (**B)** Overnight bacterial cultures in LB medium were diluted and plated on LB agar medium or V8 agar medium, and the number of colonies formed was counted. The relative CFUs on the V8 agar plate against those on the LB agar plate are shown. Data represent the mean ± SE of at least three biological repeats. One-way ANOVA with Tukey’s post hoc test *P*-values is provided (*P < 0.05).

The accumulation of Und-PP-linked ECA repeat units in the cytoplasmic leaflet of the IM induces the death of *E. coli* cells (11, 12). Therefore, we hypothesized that lipid-linked ECA repeat units are involved in the sensitivity of the *wzxE*-knockout mutant to acidic conditions. A previous study demonstrated that knockout of *wecF*, the glycosyltransferase responsible for the addition of the final FucNAc sugar to the ECA-repeat backbone trisaccharide, abolished Und-PP-linked ECA repeat units (16, 17) (Fig. 1A). Consequently, we next investigated whether the pH sensitivity of the *wzxE*-knockout strain was abolished by the knockout of *wecF* (Fig. 1A). The *wzxE*-knockout mutant showed reduced proliferative potential at low pH LB or V8, but the Δ*wzxE*Δ*wecF* double-knockout strain showed higher proliferative potential than the *wzxE* knockout mutant under these conditions (Fig. 4A). Furthermore, the Δ*wzxE*Δ*wecF* double knockout strain did not exhibit reduced CFU counts on V8 agar compared to the wild-type strain (Fig. 4B). These results suggest that Und-PP-linked ECA repeats are involved in the sensitivity of the *wzxE*-knockout strain to acidic conditions.

### Cells of the *wzxE*-knockout strain are less viable than WT cells during growth under acidic conditions

**Fig 5:**
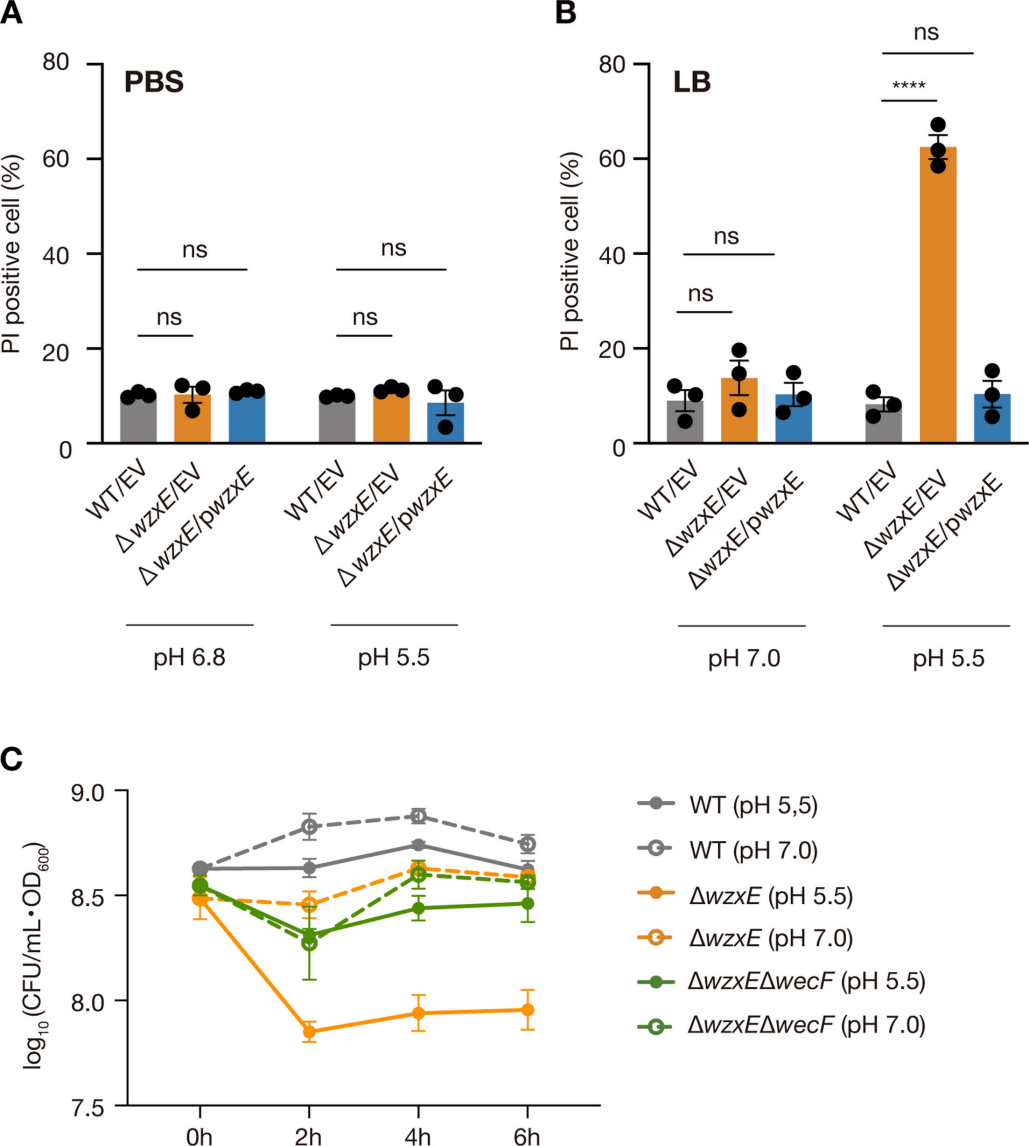
*wzxE*-knockout mutant shows an increase in dead bacteria under acidic conditions that allows bacterial growth. (**A)** *Escherichia coli* wild-type strain transformed with pMW118 (WT/EV), *wzxE*-knockout mutant transformed with pMW118 (Δ*wzxE*/EV), and *wzxE*-knockout mutant transformed with pwzxE (Δ*wzxE*/pwzxE) were treated with PBS (pH 6.8) or PBS 50 mM MES (pH 5.5) for 2 h at 37°C, followed by the addition of PI, and the fluorescence was measured using flow cytometry. Data represent the mean ± SE of three biological repeats. Two-way ANOVA with post hoc Dunnett’s test was performed (ns, *P* ≥ 0.05). (**B)** Bacteria were aerobically cultured for 2 h in LB 40 mM MOPS (pH 7.0) or LB 50 mM MES (pH 5.5) at 37°C, stained with PI, and the fluorescence was measured using flow cytometry. Data represent the mean ± SE of three biological repeats. Two-way ANOVA with post hoc Dunnett’s test *P*-values is provided (****P < 0.0001). (**C)** Overnight bacterial cultures were inoculated in LB 40 mM MOPS (pH 7.0) or LB 50 mM MES (pH 5.5) at 37°C and the OD_600_ and viable counts were measured every 2 h. The vertical axis shows the CFUs per OD_600_. Data represent the mean ± SE of three biological repeats.

To investigate the reason for the slower culture growth of *wzxE*-knockout cells, bacteria were first grown overnight in pH-unadjusted LB medium and then transferred to acidified (pH 5.5) phosphate-buffered saline (PBS) for 2 h. Cells were subsequently treated with membrane-impermeant propidium iodide, a fluorescent dye that can only label DNA/RNA when the cell envelope is compromised, allowing cytoplasmic access. Fluorescence staining with propidium iodide was then quantified via flow cytometry to determine the proportion of dead (i.e., fluorescent) cells in the population. The results revealed no significant difference in the number of viable bacteria between the WT and *wzxE*-knockout strains treated with PBS (pH 5.5) (Fig. 5A). In contrast, when bacteria were cultured for 2 h in acidified LB (pH 5.5), representing a growth-enabling condition, the number of dead bacteria was significantly higher in the *wzxE*-knockout mutant than in the WT strain (Fig. 5B). Additionally, the number of CFUs per OD_600_ was lower in the *wzxE*-knockout mutant than in the WT control strain in LB medium (pH 5.5) (Fig. 5C). The reduction in the CFUs per OD_600_ in the *wzxE*-knockout mutant was abolished by the additional knockout of *wecF* (Fig. 5C). These results suggest that wzxE knockout is less viable under acidic growth-enabling conditions due to the presence of accumulated Und-PP-linked ECA repeat units.

### The sensitivity of the *wzxE* knockout to acidic conditions depends on colanic acid biosynthesis and is rescued by overexpressed *wzxC*, a flippase for Und-PP-linked colanic acid

**Fig 6:**
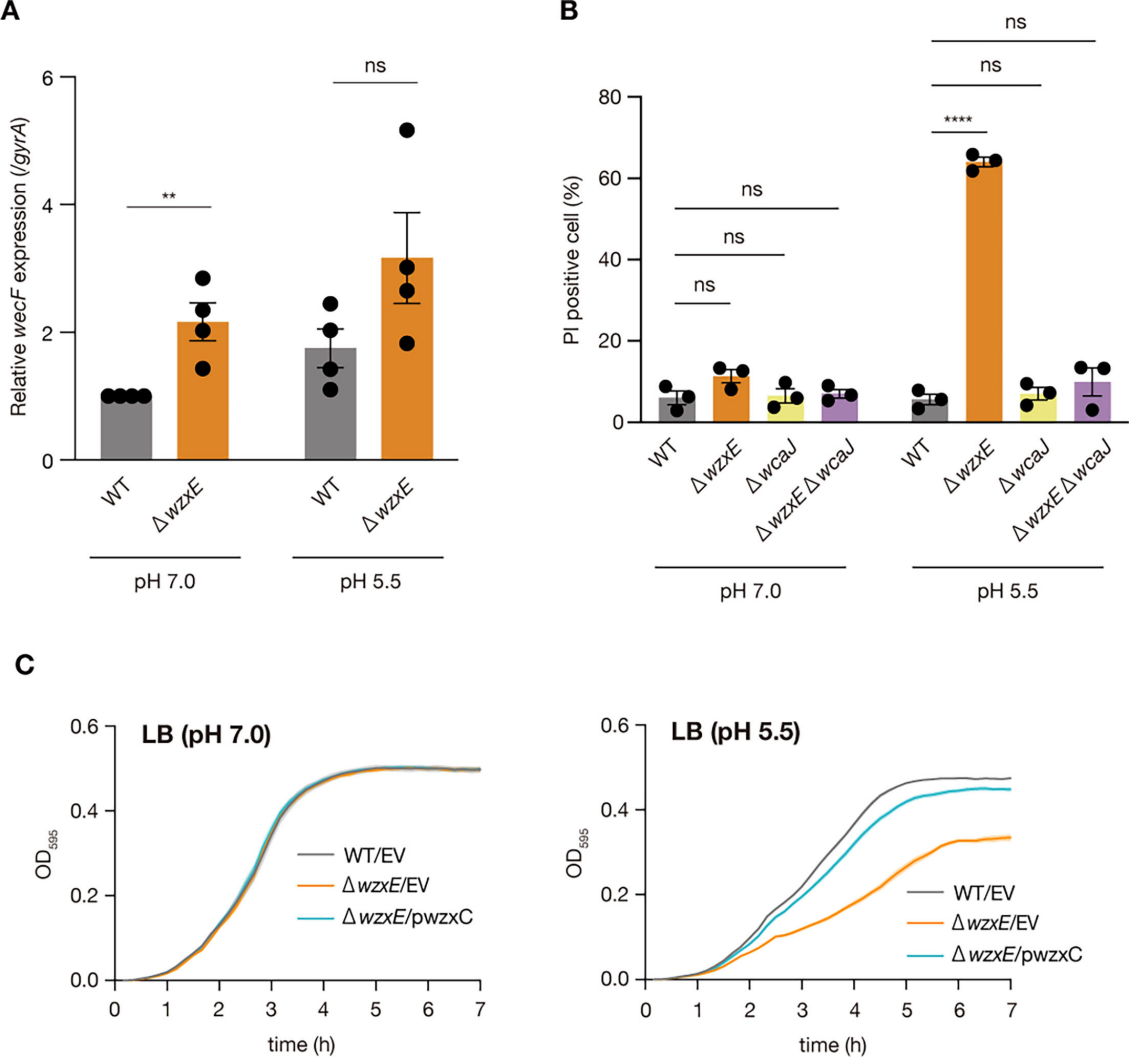
*wzxE*/*wcaJ* double knockout mutant is not sensitive to acidic conditions. (**A)** The *wecF* expression was determined using quantitative real-time PCR, with *gyrA* expression used as an internal standard. Data represent the mean ± SE of four biological repeats. Unpaired *t*-test *P*-value is provided (**P < 0.01; ns, *P* ≥ 0.05). (**B)** *Escherichia coli* wild-type strain (WT), *wzxE*-knockout mutant (Δ*wzxE*), *wcaJ-*knockout mutant (Δ*wcaJ*), and *wzxE*/*wcaJ* double knockout mutant (Δ*wzxE* Δ*wcaJ*) were aerobically cultured in LB 40 mM MOPS (pH 7.0) or LB 50 mM MES (pH 5.5) for 2 h at 37°C, and the cells were stained with PI. The fluorescence was measured using flow cytometry. Data represent the mean ± SE of three biological repeats. Two-way ANOVA with post hoc Dunnett’s test *P*-values is provided (****P < 0.0001; ns, *P* ≥ 0.05). (**C)** The *E. coli* wild-type strain transformed with pMW118 (WT/EV), *wzxE*-knockout mutant transformed with pMW118 (Δ*wzxE*/EV), and *wzxE*-knockout mutant transformed with pwzxC (Δ*wzxE*/pwzxC) were cultured in LB 50 mM MES (pH 5.5) and LB 40 mM MOPS (pH 7.0). The OD_595_ was measured for 7 h at 37°C. Data represent the mean ± SE of six biological repeats.

We hypothesized that ECA deficiency in *wzxE*-knockout cells could lead to the upregulation of ECA synthesis *via wecF* as a feedback response. Our results revealed no significant differences in *wecF* expression between the WT and *wzxE*-knockout strains at pH 5.5; however, we observed higher *wecF* expression in the *wzxE*-knockout mutant than in the WT strain at pH 7.0 (Fig. 6A). These results show that *wecF* expression is not significantly enhanced in the *wzxE*-depleted mutant under acidic conditions.

The WzxC and WzxB flippases for Und-PP-linked colanic acid and O-antigen repeat units, respectively, can flip Und-PP-linked ECA repeat units to prevent its lethal accumulation in *wzxE*-deficient strains (11–13). RcsA, a transcriptional activator of colanic acid synthesis, is upregulated in acidic environments (5, 6). Based on these findings, we hypothesized that increased synthesis of colanic acid under acidic conditions causes WzxC to be occupied by Und-PP-linked colanic acid repeat units, reducing the number of Und-PP-linked ECA repeats flipped by WzxC, thus increasing the accumulation of Und-PP-linked ECA repeat units in the *wzxE*-knockout mutant; this would render the *wzxE* mutant sensitive to acidic conditions. Experiments were conducted using a mutant strain defective in *wcaJ*, which is a glycosyltransferase required for joining the initial main-chain sugar to the lipid carrier to initiate Und-PP-linked colanic acid repeat-unit biosynthesis. The *wzxE wcaJ* double-knockout strain was also generated. The *wcaJ*-knockout and *wzxE wcaJ* double-knockout strains did not significantly increase the number of dead bacteria after growth in acidified (pH 5.5) LB, unlike the *wzxE*-knockout strain (Fig. 6B). These results suggest that lipid-linked colanic acid repeat unit synthesis is required for sensitivity to acidic conditions caused by *wzxE* deficiency.

To know whether the WzxC flippase can prevent the lethal accumulation of Und-PP-linked ECA repeat units in the *wzxE* mutant at acidic conditions, we examined the effect of *wzxC* overexpression on the growth of the *wzxE* mutant in acidic conditions. The introduction of the *wzxC* plasmid restored the growth of the *wzxE* mutant at pH 5.5 (Fig. 6C). The result suggests that overexpressed WzxC rescued the *wzxE* mutant by preventing the lethal accumulation of Und-PP-linked ECA repeat units.

### *wzxE*-knockout strain is sensitive to both low-temperature and high-salt conditions

**Fig 7:**
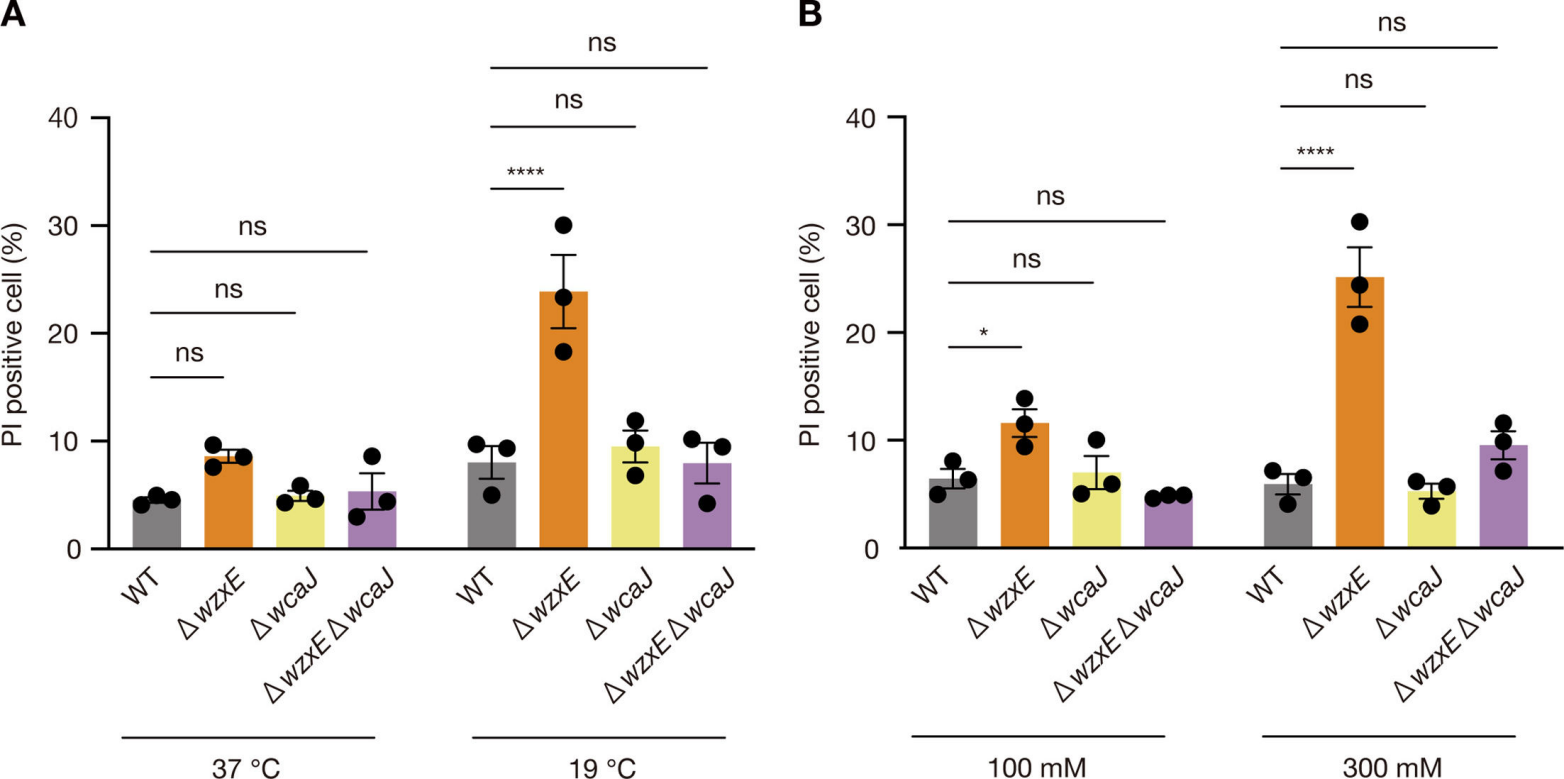
*wzxE*-knockout mutant is sensitive to low temperature and high-salt conditions. (**A)** *Escherichia coli* wild-type strain (WT), *wzxE*-knockout mutant (Δ*wzxE*), *wcaJ-*knockout mutant (Δ*wcaJ*), and *wzxE*/*wcaJ* double knockout mutant (Δ*wzxE* Δ*wcaJ*) were cultured in LB 40 mM MOPS (pH 7.0) for 24 h at 37°C or 19°C and then PI was added to stain cells. Data represent the mean ± SE of three biological repeats. Two-way ANOVA with post hoc Dunnett’s test *P*-values is provided (****P < 0.0001; ns, *P* ≥ 0.05). (**B)** *E. coli* wild-type strain (WT), *wzxE*-knockout mutant (Δ*wzxE*), *wcaJ-*knockout mutant (Δ*wcaJ*), and *wzxE*/*wcaJ* double knockout mutant (Δ*wzxE* Δ*wcaJ*) were aerobically cultured in LB 40 mM MOPS (pH 7.0) supplemented with 100 mM NaCl or 300 mM NaCl at 37°C, and then PI was added and the fluorescence was measured using flow cytometry. Data represent the mean ± SE of three biological repeats. Two-way ANOVA with post hoc Dunnett’s test *P*-values is provided (*P < 0.05; ****P < 0.0001; ns, *P* ≥ 0.05).

Colanic acid biosynthesis is induced under acidic, low-temperature, and high-salt conditions and promotes the survival of *E. coli* under these stresses (7, 8). We next examined whether the colanic acid synthesis-dependent susceptibility of the *wzxE*-knockout mutant was present not only under acidic conditions but also under low-temperature and high-salt stress. Propidium iodide staining after 24 h of incubation at 19°C (i.e., low-temperature stress) showed an increase in the number of dead bacteria in the *wzxE*-knockout mutant compared to the WT strain (Fig. 7A). The *wzxE wcaJ* double-knockout strain showed no significant increase in dead bacteria after 24 h of incubation at 19°C (Fig. 7A). Additionally, propidium iodide staining after 2 h of incubation in LB (pH 7.0) adjusted with 300 mM NaCl, representing high-salt stress, showed an increase in dead bacteria in the *wzxE*-knockout mutant compared to that in the WT strain (Fig. 7B). The *wzxE wcaJ* double-knockout strain showed no significant increase in dead bacteria in the presence of 300 mM NaCl (Fig. 7B). In contrast, when LB was adjusted with 100 mM NaCl, representing a low-salt stress condition, the *wzxE*-knockout mutant showed a slight increase in dead bacteria compared to that in the WT strain, but the difference was small compared to that in LB adjusted with 300 mM NaCl (Fig. 7B). These results suggest that the *wzxE*-knockout mutant is sensitive to low temperatures and high-salt stress in a colanic acid synthesis-dependent manner.

### *wzxC*-knockout strain is not sensitive to acidic conditions

**Fig 8:**
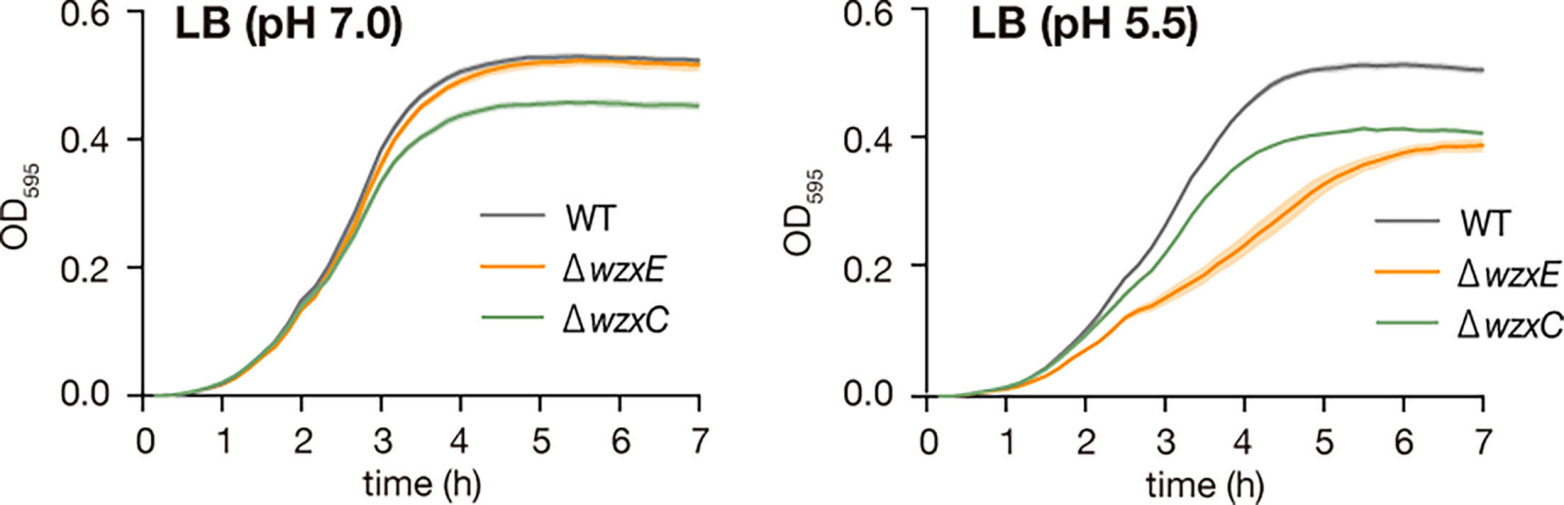
*wzxC*-knockout mutant is not sensitive to acidic conditions. *Escherichia coli* wild-type strain and the *wzxC*-knockout mutants were cultured in LB 50 mM MES (pH 5.5) and LB 40 mM MOPS (pH 7.0). The OD_595_ was measured for 7 h at 37°C. Data represent the mean ± SE of six biological repeats.

To examine whether the accumulation of Und-PP-linked repeat sugar units other than Und-PP-linked ECA repeat units increases *E. coli* sensitivity to acidic conditions, we examined whether the *wzxC*-knockout mutant that accumulates Und-PP-linked colanic acid repeat units (18) exhibits sensitivity to acidic conditions. The *wzxC*-knockout mutant showed growth defects compared to the WT strain in LB medium adjusted to pH 5.5 as well as pH 7.0 (Fig. 8). The results suggest that the accumulation of Und-PP-linked colanic acid repeat units does not induce *E. coli* sensitivity to acidic conditions, but rather induces growth defects independent of pH.

## DISCUSSION

In this study, we observed that the knockout of the Und-PP-linked ECA repeat-unit flippase *wzxE* caused *E. coli* susceptibility to acidic conditions, low temperatures, and high-salt stress. Furthermore, these stress susceptibilities depended on the ECA and colanic acid repeat-unit-building enzymes *wecF* and *wcaJ*, respectively. This study is the first to demonstrate that the Und-PP-linked ECA repeat-unit flippase gene *wzxE* is required for the resistance of *E. coli* to acidic, low-temperature, and osmotic pressure stresses in a colanic acid synthesis-dependent manner.

**Fig 9:**
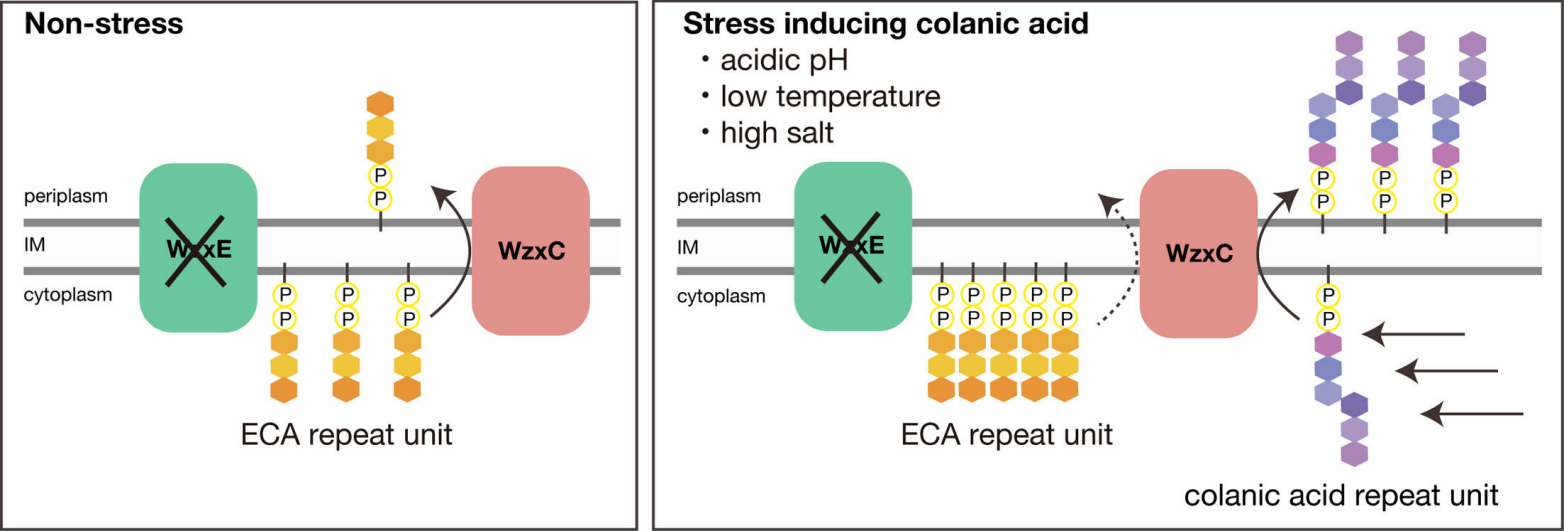
Mechanism underlying the sensitivity of the wzxE-knockout mutant against stresses that induce colanic acid synthesis. Under stress-free conditions that do not induce colanic acid synthesis, WzxC is assumed to prevent the accumulation of the lipid-linked ECA repeat unit in the *wzxE*-knockout mutant. However, under stress conditions, such as acidic, high salt, and low temperature, which induce colanic acid synthesis, WzxC is occupied by a lipid-linked colanic acid repeat unit, lipid-linked ECA repeat unit flipping by WzxC becomes less frequent, and the lipid-linked ECA repeat unit accumulates on the cytosolic side of the IM, which may induce cell death.

Based on the results of this study, we hypothesized that the increased production of colanic acid in the *wzxE*-knockout mutant under acid, low temperature, and high-salt conditions occupied WzxC and reduced the number of Und-PP-linked ECA repeat units flipped by WzxC, resulting in the lethal accumulation of Und-PP-linked ECA repeat units (Fig. 9). Under these conditions, Und-PP-linked colanic acid repeat units may also be accumulated on the IM to enhance toxicity. To clarify these points, future experiments are needed to quantify the amounts of Und-PP-linked ECA repeat units and colanic acid in the *wzxE*-knockout mutant.

We revealed no *E. coli* sensitivity to acidic conditions in the *wzxC*-knockout mutant that accumulates Und-PP-linked colanic acids and the *wecG*-, *wecF*-knockout mutant that accumulates Und-PP-linked monosaccharide or disaccharide (Fig. 1B and 8). These results suggest that the Und-PP-linked ECA repeat unit is particularly toxic at acidic conditions, or the accumulated amount of Und-PP-linked ECA repeat unit is larger than those of other repeat units at acidic conditions. The mechanism by which Und-PP-linked ECA repeat units accumulated on the cytoplasmic side of the IM causes bacterial cell death is poorly understood. Mutants that accumulate Und-PP-linked ECA, such as the *wzxE*-knockout mutant, have been reported to have an abnormal cell shape, suggesting that the lipid carrier used for peptidoglycan synthesis, undecaprenyl phosphate, is sequestered in the ECA synthesis pathway (19). Similar cell morphological abnormalities have been observed with the inhibition of O antigen or LPS core synthesis in *wzxB*- or *waaC-*knockout mutants (20). We demonstrated that the cell lysis of the *wzxE*-knockout mutant in an acidic environment occurs in a bacterial growing condition, but not in a bacterial non-growing condition using PBS (pH 5.5) and saline (pH 5.4) (Fig. 3 and 5), which is consistent with the idea that a defect in cell division due to abnormal peptidoglycan synthesis in the *wzxE*-knockout mutant causes cell lysis during bacterial growth. Future studies should investigate the underlying mechanism by which the accumulation of Und-PP-linked ECA increases *E. coli* sensitivity to acidic stress conditions, including the effects on Und-PP homeostasis, membrane defects, and peptidoglycan synthesis.

The *E. coli* BW25113 strain used in this study is derived from K-12 and does not express the O antigen. Because the O antigen flippase *wzxB* can flip the Und-PP-linked ECA repeat unit in the *E. coli* K-12 strain (11–13), the phenotype of *wzxE* deficiency may change with or without O antigen expression and serotype. O-antigen-carrying strains are of high public health importance because they are frequently isolated as causative agents of foodborne illnesses (21). Therefore, it is necessary to examine whether the mechanism described in this study applies to strains that express O antigens attached to LPS.

The decrease in the proliferative capacity and colony-forming ability of the *wzxE*-knockout mutant was more pronounced in V8 medium (pH 5.5) than in LB medium (pH 5.5) (Fig. 1 and 2). Additionally, no strict correlation was observed between the pH of the crude vegetable extract and the number of viable bacteria after culturing in the crude vegetable extract (Fig. 3). Since the osmolality of the vegetable crude extracts used in this study was not significantly different from that of the saline solution, we speculate that factors other than pH and osmolality, such as the organic acids contained in vegetables, amplify the phenotype of the *wzxE*-knockout mutant in the vegetable environment. In relation to this possibility, the *wecB*-knockout mutant that cannot synthesize ECA shows sensitivity to organic acids in the *E. coli* O157:H7 strain (22).

Compared to the *wcaJ*-knockout mutant, which is unable to synthesize colanic acid, the *wzxE*-knockout mutant showed significantly higher sensitivity to acid, low temperature, and high-salt stress (Fig. 6 and 7). Furthermore, the *wzxE*-knockout mutant exhibited reduced proliferative capacity for all vegetable extracts analyzed in this study (Fig. 3). In addition, WzxE homologs are not found in mammals, including humans, but are widely distributed and highly conserved in Enterobacteriaceae (23). The family Enterobacteriaceae contains several bacterial species that cause food poisoning, including *Shigella*, *Salmonella*, and *Yersinia* species. These findings suggest that the development of inhibitors against WzxE may lead to new food poisoning prevention agents that break the bacterial cycle between plants and humans by inhibiting the growth of *E. coli* in acidic environments such as the plant and intestinal tracts.

## MATERIALS AND METHODS

### Bacterial strains and culture conditions

*Escherichia coli* BW25113 and its gene-knockout mutants were cultured on LB agar medium, and the colonies were aerobically incubated at 37°C in LB liquid medium. *Escherichia coli* strains transformed with pMW118 were cultured on LB agar medium containing 100 µg/mL ampicillin. The bacterial strains and plasmids used in this study are listed in Table 1.

**TABLE 1:** Bacterial strains and plasmids used in this study

Strain or plasmid	Genotype or characteristics	Reference
Strains		
BW25113	*rrnB*, Δ*lacZ*4787, *HsdR*514 Δ(*araBAD*)567, Δ(*rhaBAD*)568, *rph-1*	NBRC
JW3758	BW25113 Δ*wecA*; Km^r^	Baba et al*.* (24)
JW5600	BW25113 Δ*wecB*; Km^r^	Baba et al*.* (24)
JW5599	BW25113 Δ*wecC*; Km^r^	Baba et al*.* (24)
JW5597	BW25113 Δ*wecD*; Km^r^	Baba et al*.* (24)
JW3765	BW25113 Δ*wecE*; Km^r^	Baba et al*.* (24)
JW5596	BW25113 Δ*wecF*; Km^r^	Baba et al*.* (24)
JW3770	BW25113 Δ*wecG*; Km^r^	Baba et al*.* (24)
JW3766	BW25113 Δ*wzxE*; Km^r^	Baba et al*.* (24)
JW3766ML	BW25113 Δ*wzxE*; markerless	This study
JW5601	BW25113 Δ*wzzE*; Km^r^	Baba et al*.* (24)
JW3597	BW25113 Δ*waaL*; Km^r^	Baba et al*.* (24)
JW2031	BW25113 Δ*wzxC*; Km^r^	Baba et al*.* (24)
SY00001	BW25113 Δ*wzxE-wecF*; Km^r^	This study
JW2032	BW25113 Δ*wcaJ*;Km^r^	Baba et al*.* (24)
SY00002	BW25113 Δ*wzxE*; markerless, Δ*wcaJ*; Km^r^	This study
JM109	Host strain for cloning	Takara Bio
Plasmids		
pMW118	Low-copy-number plasmid; Amp^r^	Nippon Gene
pMW118-wzxE	pMW118 with *wzxE*; Amp^r^	This study
pMW118-wzxC	pMW118 with *wzxC*; Amp^r^	This study
pKD46	A temperature-sensitive plasmid expressing RED recombinase; Amp^r^	CGSC, Datsenko et al*.* (25)
pCP20	A temperature-sensitive plasmid expressing FLP recombinase; Amp^r^	CGSC Datsenko et al*.* (25)

### Preparation of V8 medium

A slightly modified version of a previously published method (26) was used. Specifically, 340 mL of V8 vegetable juice (Campbell) was stirred with 5 g of CaCO_3_. The mixture was then centrifuged at 2,590 × *g* for 10 min, and the supernatant was diluted fivefold with Milli-Q water and autoclaved to obtain V8 medium. V8 agar medium was prepared by adding 20 g/mL of agar to the V8 medium, followed by autoclaving. The pH-adjusted medium was prepared by adding buffer (pH 5.5, 50 mM MES; pH 7.0, 40 mM MOPS; pH 8.5, 50 mM tricine) and adjusting the pH using NaOH.

### Genetic manipulation

The *wzxE* and *wcaJ* gene-knockout mutants were generated *via* transduction with P1 phage *vir* using JW3766 or JW2032 as phage donors and BW25113 as the recipient strain. The *wzxE*-depleted mutant was transformed with pCP20, resulting in a marker-less *wzxE-*depleted mutant. Transduction with P1 phage *vir* was performed using the marker-less *wzxE-*knockout mutant as a recipient strain and JW2032 as a phage donor to generate the *wzxE*/*wcaJ* double-knockout mutant. To construct the complementation plasmid, a DNA fragment containing *wzxE* was amplified using PCR with BW25113 genomic DNA as a template and oligonucleotide primer pairs. The DNA amplification product was inserted at the XbaI and HindIII sites of pMW118 to obtain pMW118-wzxE. To generate the *wzxE*/*wecF* double-knockout mutant, the *wzxE* and *wecF* genes were deleted from BW25113 cells using the one-step inactivation method (25).

### Growth curve

Briefly, 2 µL of an overnight culture of *E. coli* was added to 100 µL of the medium in a 96-well microplate and sealed with a plastic cover. The OD_595_ was measured at 10 min intervals for 7 h in a microplate reader (Thermofisher) with shaking at 37°C.

### Colony-forming ability

Dilutions of overnight cultures of *E. coli* were spread on agar medium, and the number of colonies formed after incubation at 37°C was counted. The incubation times differed depending on the medium used: overnight for LB, LB 40 mM MOPS (pH 7.0), LB 50 mM tricine (pH 8.5), V8 40 mM MOPS (pH 7.0), and V8 50 mM tricine (pH 8.5); two nights for LB 50 mM MES (pH 5.5); and three nights for V8 50 mM MES (pH 5.5).

### Determination of viable bacteria in plant crude extracts

Crude vegetable extracts were prepared by crushing each vegetable and filtering it through gauze. Overnight bacterial cultures were added to 100 µL of vegetable crude extract supplemented with ampicillin and incubated at 25°C for 24 h. The culture medium was then serially diluted and applied to the LB agar medium, and the number of CFUs was measured after overnight incubation. The same procedure was performed for samples that were incubated overnight at 25°C in 0.9% NaCl as controls.

### Flow cytometry

A slightly modified version of a previously published method (27) was used for the analysis. Bacterial overnight culture (1 mL) was centrifuged at 4,000 × *g* for 5 min, and bacterial cell pellets were suspended in PBS or PBS 50 mM MES (pH 5.5) to adjust the OD_600_ and incubated at 37°C for 2 h. Alternatively, overnight cultures were inoculated in LB 50 mM MES (pH 5.5) or LB 40 mM MOPS (pH 7.0), incubated at 37°C for 2 h, and suspended in PBS to adjust the OD_600_. Subsequently, propidium iodide (7.5 µg/mL) was added, and fluorescence was measured using flow cytometry after incubation at 37°C for 15 min in the dark. For cold stress, the bacteria were incubated in LB 40 mM MOPS (pH 7.0) at 19°C for 24 h, stained with propidium iodide, and analyzed using flow cytometry. For high-salt stress, bacteria were cultured for 2 h in LB 40 mM MOPS (pH 7.0) adjusted to NaCl 100 mM or 300 mM, propidium iodide staining, and then measured using flow cytometry.

### Quantitative PCR

The total RNA from *E. coli* was analyzed using a slightly modified version of a previously reported method (28, 29). *E. coli* cells were cultured in 5 mL of LB 50 mM MES (pH 5.5) or LB 40 mM MOPS (pH 7.0) for 2 h. Following incubation, 1.8 mL of the culture was mixed with 200 µL of ethanol containing 5% phenol by vortexing and centrifuged at 21,500 × *g* for 2 min, and the precipitates were frozen in liquid nitrogen. The bacteria were resuspended in 200 µL of lysis buffer (TE buffer, 1% lysozyme, 1% SDS) and incubated at 65°C for 2 min. RNA was extracted using the RNeasy Mini Kit (QIAGEN, Tokyo, Japan), treated with DNase I, and used as a template for cDNA synthesis using SuperScript II reverse transcriptase and a random hexamer. Quantitative PCR was performed using the synthesized cDNA as a template using oligonucleotide primer pairs (Table 2) and KOD SYBR pPCR Mix (TOYOBO, Osaka, Japan). QuantStudio 3 (Thermo Fisher Scientific, Tokyo, Japan) was used for the assay, and gyrA was used as the internal control.

**TABLE 2:** Primers used in this study

Primer name and purpose	Primer sequence
Amplification of *wzxE*	
wzxE_F_XbaI	TCTTCTAGAAATCATGGCGGTGTTTCATT
wzxE_R_HindIII	AGAAGCTTGAGGGATATCCGATCCCAGT
wzxC_F_XbaI	TCTTCTAGACCTGACGGTGTTCAAAGGTT
wzxC_R_HindIII	AAGAAGCTTAAATTTGATGCCAGGTGAGG
One-step inactivation	
wzxE_H1_P1	ACGGTAATTGCGACTTTGTTGAACTACTTTTCCTGATATGGTGTAGGCTGGAGCTGCTTC
wecF_H2_P2	TCATGCGACCTCCCTGGCGGCAATCGCCAACGCCCGCTGCCATATGAATATCCTCCTTAG
qPCR	
wecF_F	CAACGTGAAGGGAAAATGACC
wecF_R	CATCGGCACCACCACTTTTAC
gyrA_F	AGTCTTCTGTCCGTGCGATG
gyrA_R	GTTTTGCGTTGCGGTGAG

### Statistical analysis

All statistical analyses were performed using GraphPad PRISM software.
